# Supplementary material for: High fat diet worsens Alzheimer’s disease-related behavioral abnormalities and neuropathology in APP/PS1 mice, but not by synergistically decreasing cerebral blood flow
Source: Sci Rep. 2020 Jun 18;10:9884. doi: 10.1038/s41598-020-65908-y (PMC7303150; doi:10.1038/s41598-020-65908-y)
Supplement: Supplementary file 1 — Supplementary information. [file 41598_2020_65908_MOESM1_ESM.pdf]

Supplementary Material for: High fat diet worsens Alzheimer's disease-related behavioral abnormalities and neuropathology in APP/PS1 mice, but not by synergistically decreasing cerebral blood flow

Authors:

Oliver Bracko[1], Lindsay K. Vinarcsik[1], Jean C. Cruz Hernández[1], Nancy E. Ruiz-Uribe[1], Mohammad Haft-Javaherian[1], Kaja Falkenhain[1], Egle M. Ramanauskaite[2], Muhammad Ali[1], Aditi Mohapatra[1], Madisen Swallow[1], Brendah N. Njiru[1], Victorine Muse[1], Pietro E. Michelucci[2], Nozomi Nishimura[1], and Chris B. Schaffer[1*]

Affiliation:

[1]Meinig School of Biomedical Engineering, Cornell University, Ithaca, NY, USA

[2]Human Computation Institute, Ithaca, NY, USA

Corresponding author: cs385@cornell.edu

Supplemental Figures:

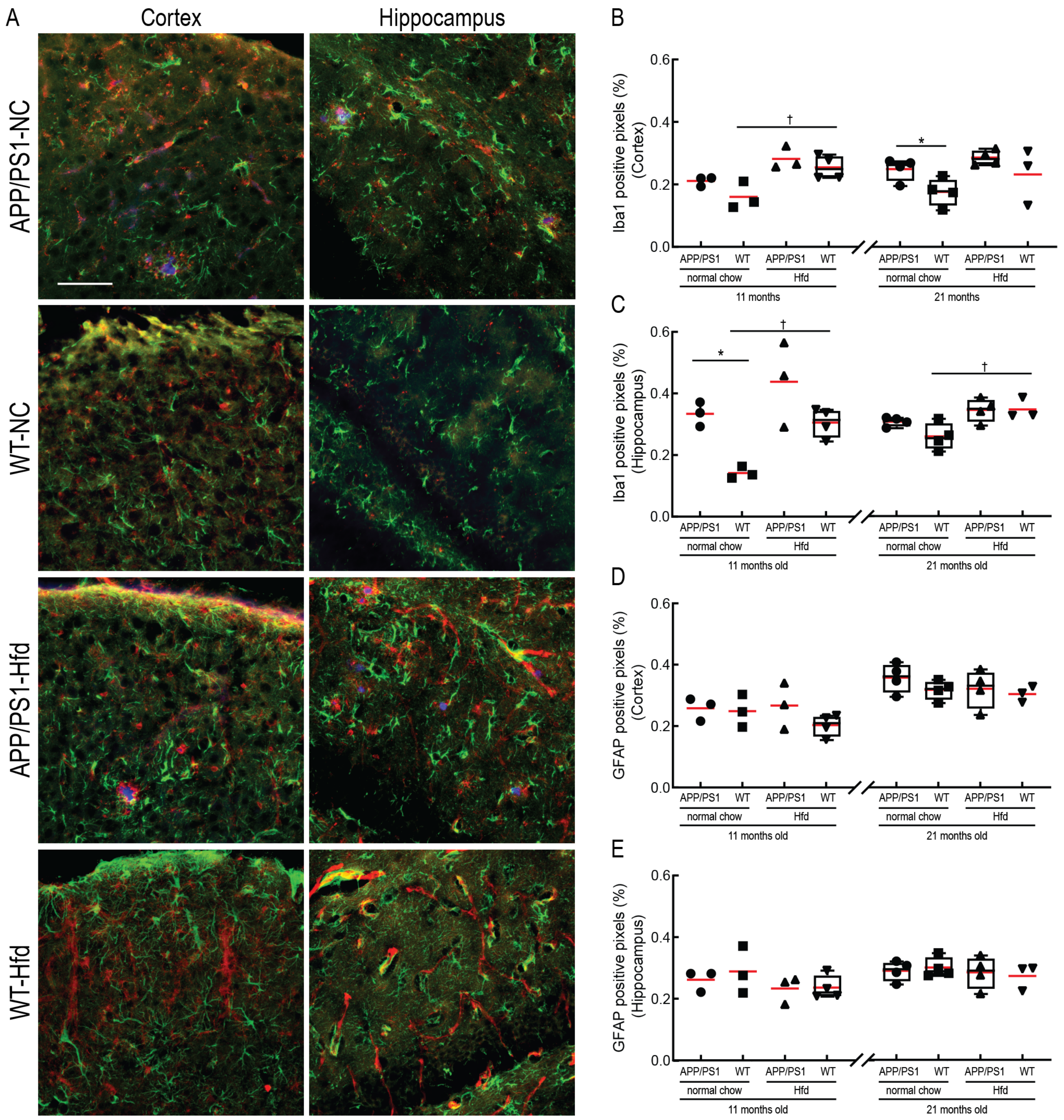

Supplementary Figure 1: Immunostaining of cortex and hippocampus in APP/PS1 and WT mice on a Hfd or normal chow. (A) Confocal images of cortical (left) and hippocampal (right) regions in APP/PS1 and WT mice on a normal chow (top) or the Hfd (bottom). Anti-GFAP for astrocytes (green), anti-IBA1 for microglia (red), Methoxy-X04 for amyloid plaques (blue). Scale bar indicates 50 µm. (B-E) Fraction of pixels positive for anti-IBA1 in the cortex (B) and hippocampus (C), and fraction of pixels positive for anti-GFAP in the cortex (D) and hippocampus (E) of APP/PS1 and WT mice on a Hfd or normal chow at 11 and 21 months of age. Animal numbers for all measurements — 11 months: APP/PS1-NC: n=3; WT-NC: n=3; APP/PS1-Hfd: n=3; WT-Hfd: n=4; 21 months: APP/PS1-NC: n=4; WT-NC: n=4; APP/PS1-Hfd: n=4; WT-Hfd: n=3; * $p<0.05$ between genotypes (APP/PS1 vs. WT); $p<0.05$, $p<0.01$ between diets (Hfd vs. NC); one-way ANOVA with post-hoc pair-wise comparisons using Dunn's multiple comparison test.

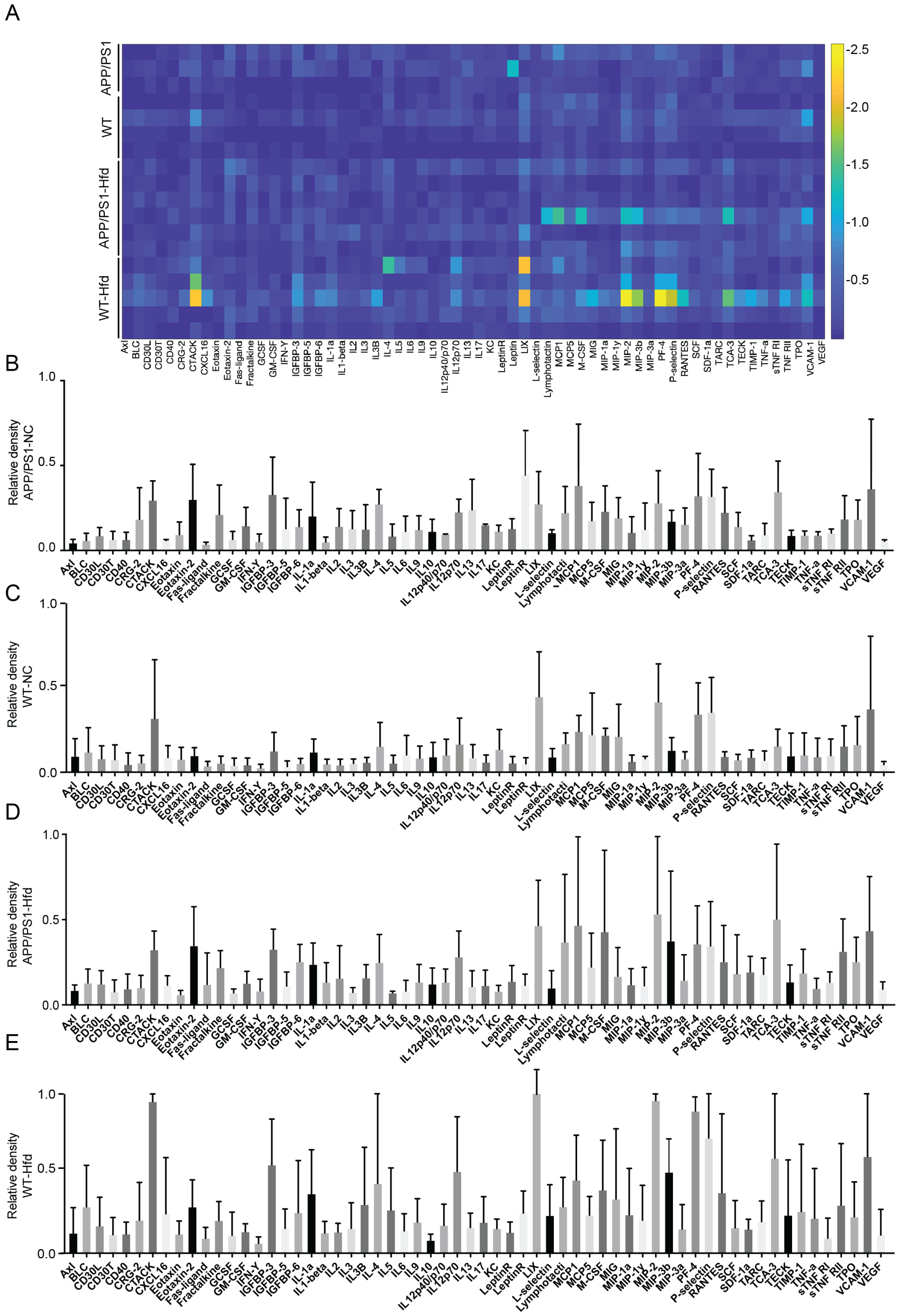

Supplementary Figure 2: Comparison of cytokine profile array data for WT and APP/PS1 mice on the Hfd or normal chow. (A) Heatmap of the brain cytokine levels for all WT and APP/PS1 mice on the Hfd or normal chow at 21 months of age. The color bar calibrates the fold changes of expression, relative to manufacturer internal controls. (B-E) Groupwise averaged relative cytokine expression for (B) APP/PS-NC: n=3; (C) WT-NC: n=4; (D) APP/PS1-Hfd: n=6; (E) WT-Hfd: n=5.

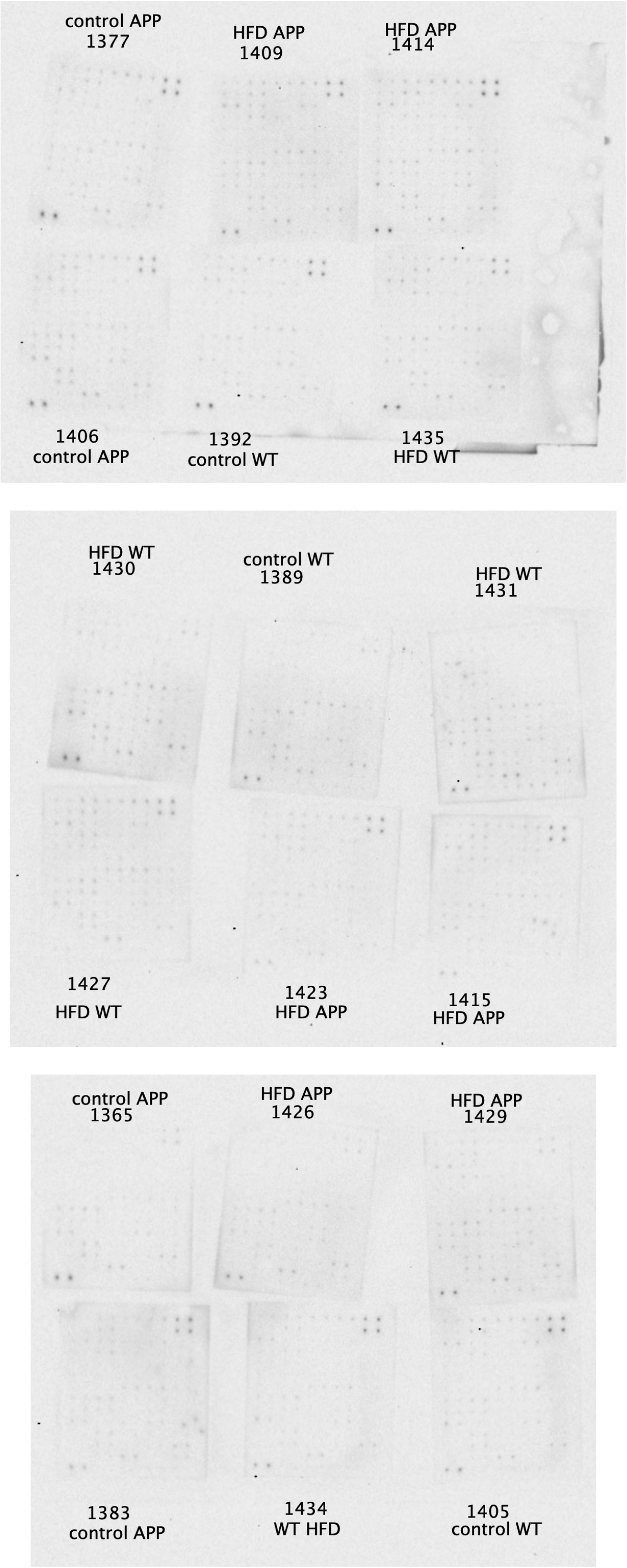

Supplementary Figure 3: Raw images of cytokine arrays of APP/PS1 and WT mice on a Hfd or normal chow. Raw images with an exposure time of 2 minutes for each individual membrane. Indicated are the animal numbers and experimental group.

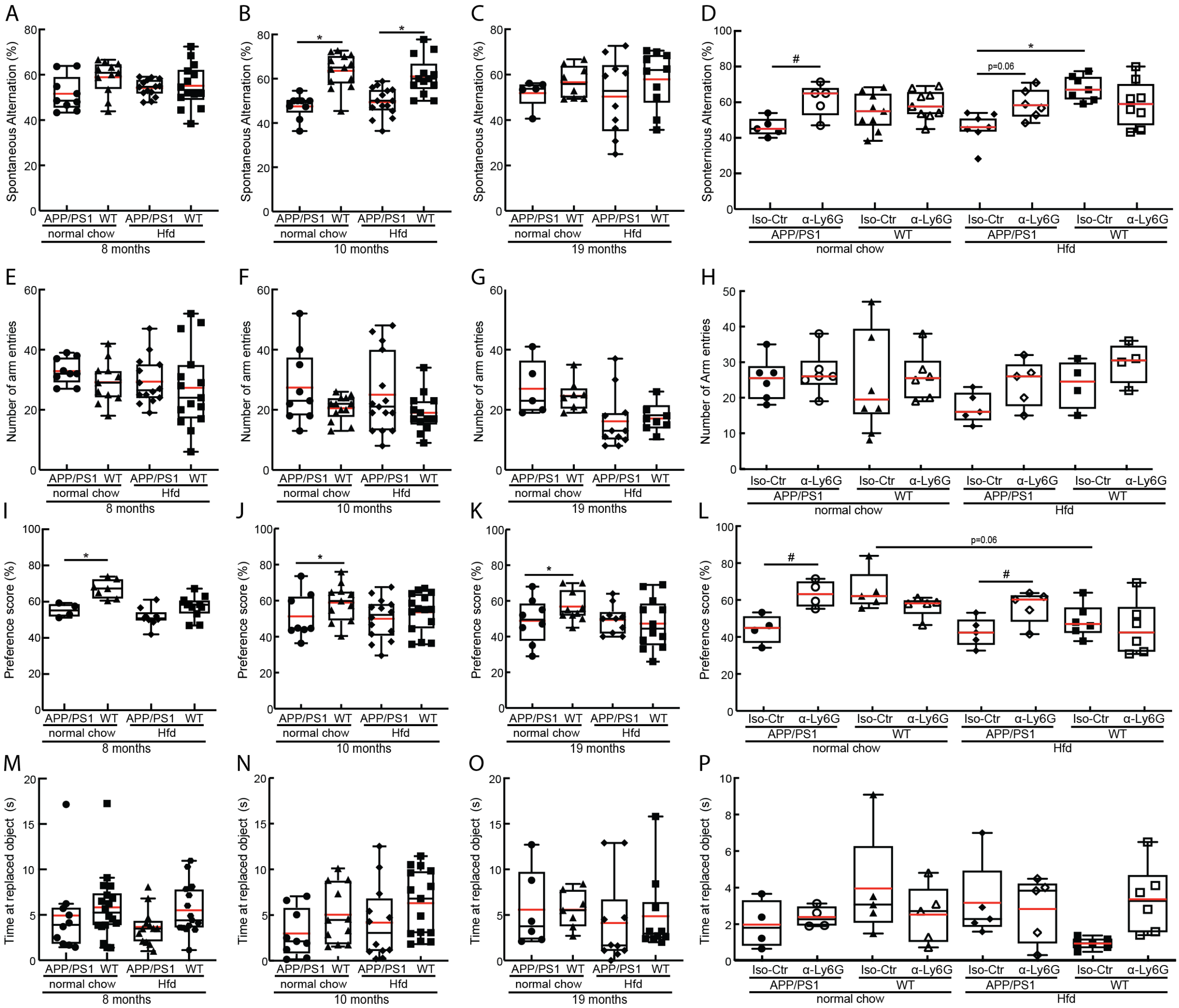

Supplementary Figure 4: Mouse-by-mouse data for short-term memory tests. (A-D) Box plots of the spontaneous alternation score and (E-G) number of arm entries from Y-maze experiments, taken at baseline for mice at 8, 10, and 19 months of age, and taken 24 hrs after α-Ly6G or Iso-Ctr antibody administration at the 19 month time point (4 mg/kg animal weight, intraperitoneal). (I-L) Box plots of the preference score and (E-G) number of arm entries from the object replacement task, taken at baseline for mice at 8, 10, and 19 months of age, and taken 24 hrs after α-Ly6G or Iso-Ctr antibody administration at the 19 month time point (4 mg/kg animal weight, intraperitoneal. 8 and 10 months: APP/PS1-NC: n=6; WT-NC: n=9; APP/PS1-Hfd: n=10; WT-Hfd: n=11; 19 months: APP/PS1-NC α-Ly6G: n=5; APP/PS1-NC Iso-Ctr n=4; WT-NC α-Ly6G: 7; WT-NC Iso-Ctr: 6; APP/PS1-Hfd α-Ly6G: n=5; APP/PS1-Hfd Iso-Ctr: n=5; WT-Hfd α-Ly6G: n=4; WT-Hfd Iso-Ctr: n=5. *p<0.05 between treatment groups (Ly6G vs. Iso-Ctr); # p<0.05 between treatment groups (Ly6G vs. Iso-Ctr), Kruskal-Wallis one-way ANOVA with post-hoc wise comparisons using Dunn's multiple comparison test.

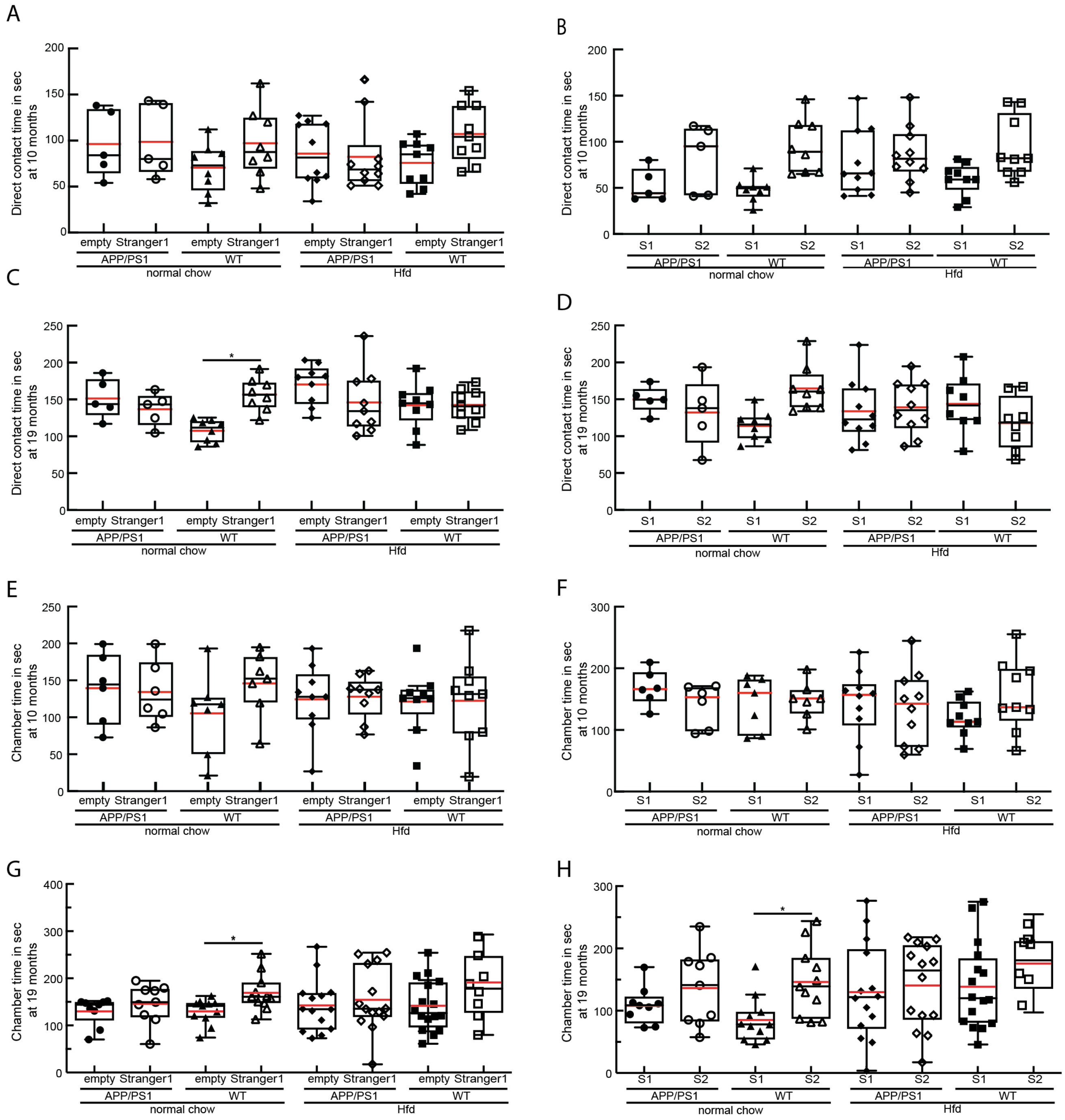

Supplementary Figure 5: Mouse-by-mouse data for the 3-chamber social interaction and social novelty test. (A-D) Box plots of direct contact time with Stranger 1 vs the empty chamber in the sociability test (A and C) and with Stranger 2 vs Stranger 1 (B and D) from the social novelty test for APP/PS1 and WT mice on a Hfd or normal chow at 10 months (A and B) or 19 months (C and D) of age. (E-H) Box plots of chamber time with Stranger 1 vs the empty chamber in the sociability test (A and C) and with Stranger 2 vs Stranger 1 (B and D) from the social novelty test for APP/PS1 and WT mice on a Hfd or normal chow at 10 months (A and B) or 19 months (C and D) of age.

Animal numbers for all measurements — 10 months: APP/PS1-NC: n=9; WT-NC: n=11; APP/PS1-Hfd: n=13; WT-Hfd: n=13; 19 months: APP/PS1-NC: n=5; WT-NC: n=8; APP/PS1-Hfd: n=10; WT-Hfd: n=11; *$p<0.05$ and **$p<0.01$; one-way ANOVA with Holm-Šídák multiple comparisons correction.

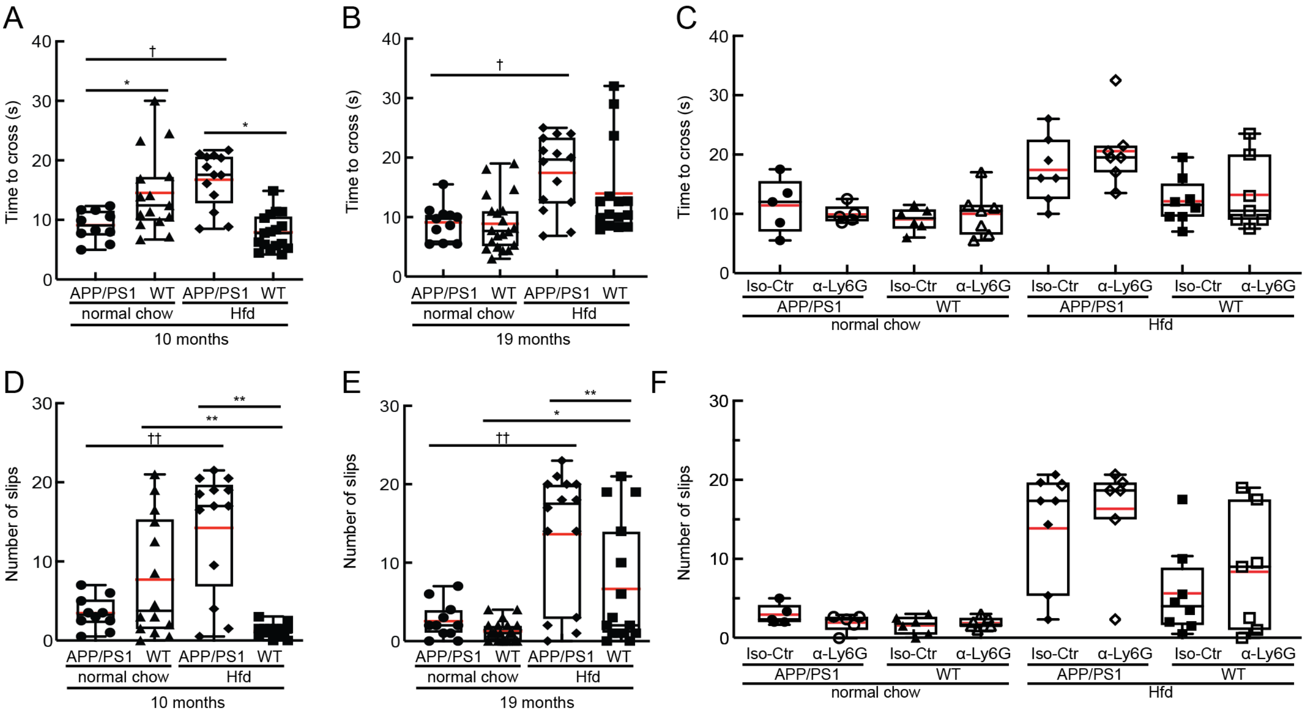

Supplementary Figure 6: Mouse-by-mouse data for the balance beam walk test of sensory-motor function. (A-B) Box plots of the time to cross the beam for the balance beam test for mice at 10 and 19 months of age, and (C) time to cross taken 24 hrs after α-Ly6G or Iso-Ctr antibody administration at the 19 month time point (4 mg/kg animal weight, intraperitoneal). (D-E) Box plots of the number of hindpaw slips in APP/PS1 and WT mice on a Hfd or normal chow at 10 and 19 months of age, and (F) number of hindpaw slips taken 24 hrs after α-Ly6G or Iso-Ctr antibody administration at the 19 month time point (4 mg/kg animal weight, intraperitoneal). Animal numbers for all measurements — 10 months: APP/PS1-NC: n=6; WT-NC: n=9; APP/PS1-Hfd: n=10; WT-Hfd: n=11; 19-months: APP/PS1-NC α-Ly6G: n=5; APP/PS1-NC Iso-Ctr n=4; WT-NC α-Ly6G: n=7; WT-NC Iso-Ctr: n=6; APP/PS1-Hfd α-Ly6G: n=5, APP/PS1-Hfd Iso-Ctr: n=5; WT-Hfd α-Ly6G: n=4; WT-Hfd Iso-Ctr: n=5. * $p<0.05$ and ** $p<0.01$ between genotypes (APP/PS1 vs. WT); † $p<0.05$, ††$p<0.01$ between diets (Hfd vs. NC), one-way ANOVA with post-hoc pair-wise comparisons using Dunn's multiple comparison test.

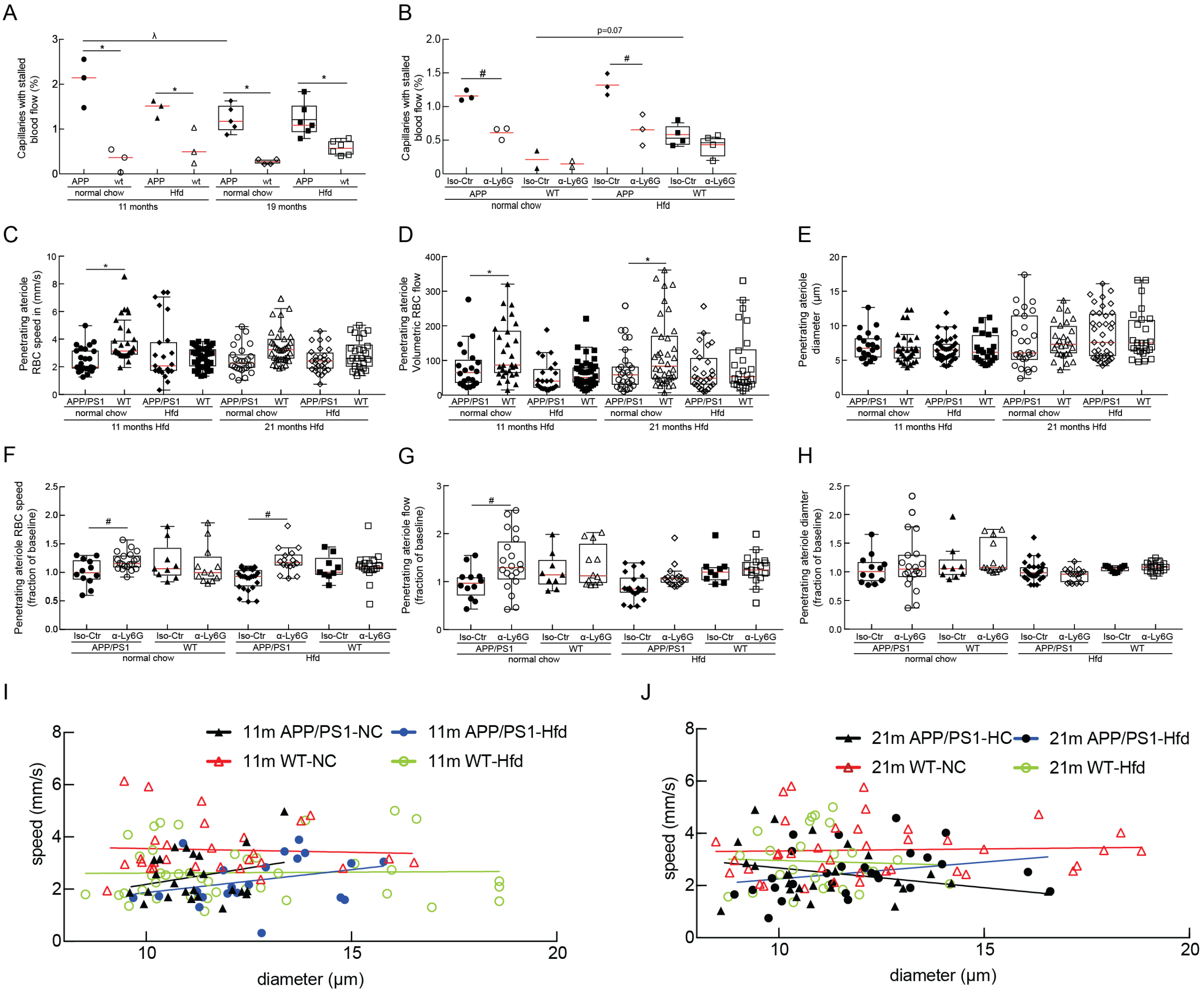

Supplementary Figure 7: Mouse-by-mouse data for the fraction of capillaries with stalled blood flow and for penetrating arteriole blood flow speeds in APP/PS1 and WT mice on normal chow or Hfd, before and after treatment with α-Ly6G or Iso-Ctr antibodies. (A) Box plot of the fraction of capillaries with stalled blood flow from 11 and 21 month old APP/PS1 and WT mice on the Hfd or normal chow. Animal numbers for all measurements — 11 month: APP/PS1-NC: n=3; WT-NC: n=3; APP/PS1-Hfd: n=3; WT-Hfd: n=3; 21 months: APP/PS1-NC: n=6; WT-NC: n=6; APP/PS1-Hfd: n=6; WT-Hfd: n=6. * $p<0.05$ between genotypes (APP/PS1 vs. WT); # $p<0.05$ between treatment groups (Ly6G vs. Iso-Ctr); ^ $p<0.05$ between animal ages; Kruskal-Wallis one-way ANOVA with post-hoc pair-wise comparisons using Dunn's multiple comparison test. (B) Box plot of the fraction of capillaries with stalled blood flow ~1 hour after α-Ly6G or Iso-Ctr antibody administration (4 mg/kg animal weight, intraperitoneal) for 21 month old mice. Animal numbers for all measurements — APP/PS1-NC α-Ly6G: n=3; APP/PS1-NC Iso-Ctr n=3; WT-NC α-Ly6G: 3; WT-NC Iso-Ctr: 3; APP/PS1-Hfd α-Ly6G: n=3; APP/PS1-Hfd Iso-Ctr: n=3; WT-Hfd α-Ly6G: n=3; WT-Hfd Iso-Ctr: n=3. * $p<0.05$ for comparisons between genotypes (APP/PS1 vs. WT), Kruskal-Wallis one-way ANOVA with post-hoc pair-wise comparisons using Dunn's multiple comparison test. Box plots of (C) RBC flow speed, (D) volumetric blood flow, and (E) vessel diameter from 11 and 21 month old APP/PS1 and WT mice on the Hfd or normal chow. Penetrating arteriole numbers (mice numbers) for all measurements — 11 months: APP/PS1-NC: n=26 (3); WT-NC: n=37 (3); APP/PS1-Hfd: n=27 (3); WT-Hfd: n=26(3); 21 months: APP/PS1-NC: n=33 (6); WT-NC: n=22 (5); APP/PS1-Hfd: n=42 (7); WT-Hfd: n=26 (6). * $p<0.05$ between genotypes (APP/PS1 vs. WT), Kruskal-Wallis one-way ANOVA with post-hoc pair-wise comparisons using Dunn's multiple comparison test. (F) RBC flow speed, (G) volumetric blood flow and (H) vessel diameter, all expressed as a fraction of the baseline value, for 21 month old APP/PS1 and WT mice taken 24 hrs after α-Ly6G or isotype control antibody administration. Penetrating arteriole numbers (mice numbers) for all measurements — APP/PS1-NC α-Ly6G: n=21(3); APP/PS1-NC Iso-Ctr n=12 (3); WT-NC α-Ly6G: 13 (3); WT-NC Iso-Ctr:9 (2); APP/PS1-Hfd α-Ly6G: n=18 (4); APP/PS1-Hfd Iso-Ctr: n=24 (3); WT-Hfd α-Ly6G: n=15 (3); WT-Hfd Iso-Ctr: n=11 (3). # $p<0.05$ between treatment groups (Ly6G vs. Iso-Ctr), Kruskal-Wallis one-way ANOVA with post-hoc pair-wise comparisons using Dunn's multiple comparison test. (I and J) Scatter plots of blood flow speed vs. vessel diameter for penetrating arterioles in APP/PS1 and WT mice fed the Hfd or normal chow at (I) 11 months and (J) 21 months of age.

Catchers who analyzed the High Fat Diet dataset

This is a list of online volunteers (aka "Catchers") who played Stall Catchers to analyze Alzheimer's research data for the Schaffer-Nishimura Laboratory in the Biomedical Engineering Department, Cornell University. The analysis provided by these Catchers was used to answer research questions concerning the role of cardiovascular risk factors in brain capillary stalling, which has been linked to Alzheimer's disease.

caprarom, lscatcher, christiane, gcalkins, Carol_aka_Mema, Tom_Adams, bartb, Michael_Landau, elAmber, Pierpaolo, GnabGib, annettei, sachambers, glol, Jamie_Lin, stephencpcd, PeterBullen, Badstallsbadbad, suzing, sean4046, halwebb, Jim, FlowFinder, MaisyMoo92, pdurr, nalluu, Karli, ababbie, MICKRUBY, Mile, MonicaPz, aussiemomma, LoBaby, austinross, monkey, Kogrady, kpatel11, badhop55, GENBOZ, achauhan1, vlimbachiya, JustinKeller2611, Sicilian, tgrap, QueenV, ChetBflo, connerposton, michaelcolombo, Vaidas_Kalpokas, Plenum, brienhodge, Michael_DeMartino, wynnhotchkiss, pgollamudi, njoshi1, gamador, jfrancis, Salish, japayt, asridhar1, th0mms3n, isaac44, AnimePersonRylee82, adavis5, rparikh, chairstar, amodi, solojmartin, celkhatib, christophermadison, camipancakelikestosneeze6, Uganalandia, Sherman_Peabody, Pezerker, RickGuar, gyfteascatlikestowin5, Jian_Bo, Zach, Wildkarrde55, FC, Maisfeeka, Laurynas_Bud, elFeladucko, c.f., jordanl52600, AwesomeMode.21, sjagarlamudi, Lar_N_Tiff, Mary4g, landosmith101, fimbriano, Mae, Brittany_Ryan, scatcher, janessacruz, Wendy_Gonzales, Emily_Priddy, Dawn_Grib, jonahposton, Arimantas, Paul_Cuthbertson, justacea, slsanders, Heath, SkylerJae56, Ladysteph, Nyx, Estelle_Angelinas, Rstevens, hannahboroff20, JoeKerr, joseph_Gattuso, Batwoman, mochness, Ami, Amanda_Looper, s_suresh, BillT84SSMD, eboza0304, to87, Carolyn_Bishop, Thor_Doss, rabTAI, David_Orrego, Parker_Owens, tsunemoto, The_Leech, abish0434, Ismail, zknig5445, marksurfs, TeresaTurley, 3KidCircus, Ashley_Backus, demetriowens, mcfar9396, John Ramos, birdsofafeather, SophiaK, Nikos, dwheeler904, ryan page, jenniepoo2000, Samuel_Mallon, vonmatter, mr99554, 9aarmuth9, 2ekky77k, bmukul30, aclab2851, JosieLikesAnime, anthony_coan, 21aysiab, tajarimooe, AllisunD143, CapriCorn, Torrance_Stephens, klaus.thenmayer, an, Evaldas, sarinozi, Vani Parthiban, seethruw, Peter_Eberhart, Darius_NeZinovaS, Marius_Vitkauskas, amirahrichmond, Sarah_Gulbranson, theblue262, parrottgreg, Adrienne_Tapia, jboyer, April_Baronas, Jacob, Redxtreme, jalenjanz-mcknight, James_James, seplute/HQ, ahard1743, kmolnar, Nitedula, mbaker11, Dareae_Thomas, KathleenScully, donpatte, Kien-Pong, astar1351, cericso, bjens4534, jeromehaley, andama a, Tomas_Totorius, robedleg, Ignaskarb, pietro/HQ, livhelfer, mord0239, MTOgress, vi.zo, Aaliyah.O, ayoung45, Plaidhand, vinnylaxy, miho_naito, Mantas_Petkelis, keshawnmcgeee, Ashwin1233, marquelllmoore, Laurynas_GiriÅ«nas, vpatsa, davionmcknight, Kirsty_Blackman, bgunnam, Leah_Schumacher, wii, Michelle_Grealish, SC_Admin, cpoar9559, YourPalRoss, DaVilA_, Benjamin_Missbach, otuitt, NizariaGiles, courtneyhoene, SearsonAlex, Zinc, mmilligan, mew, FastCureBoi, tgriz9923, andrey373, ILEMGI, Stall, Influenza, Dalius_Dulevicius, BrendahNjiru, kcoralee, DrVicenteS, Bean01, DragonRider841, marrianocoman, mshyer, Schavan, Parthiban_Jayaraman, GonÃ§alo_Catarino, JuanAmaro, Mary_Ann, noellehubbard, thisisjaid, Nicole_Huddleston, midaco, joshua951, gavin9336, avcoyle0013, Chrisbusse, EmilÄ—, SB, Jevgenija_Å½eldakova, AnythingWithJ, Noah_Richardson, Stallcatcher, Jukuda, lucyruiz, Els, AnjeloA89427, Hayli_Sanders, Naomee_Ryana, Lise_Larsen, Patrick_Gornik, JaCaLambert, Laura_Lyster-Mensh, jtown9958, Oyekola Oluyimika Oloyede, Agustina, yage123, avogel, linloth, Gabrielle_Post, Dominykas_dumbre, Brooke, Jolande_Rommens-Musquetier, Tyler_Williams, ldobb, oranele, Patrick_Lehner, Jacob_Burton, IvanauskasGytis, CR, jmullen1, haji0638, aloha2you, rrutl1967, ZouthZtar, Sylvkain, Lparames.23, WHATAMIDOINGHERE, genille6416, zezzler, RaptorKaz, awebb3297, Brody_Mette, karlamedrano3, lpahl1179, Darina, Nathan_Peterson, Susan_Fox, Deb, cmars4809, evan0475, jaydinsmith, tris_coll, Rachel_Carol, Sessavon, nomarie, NoBlock66, Matty_the_ninja, smedukonduru, paige0110, Daniel_Kusmaul, Ryan_Tse, Fluffet, Deannaunderwood, piustran992,

ukhodakiya, emirom604, TJrichmond, Brandi_Celia, Tim_Morris, Liz_Abeth, Trisarahtops, HMcCullen, Caroline_Strang, omarrionalford, venus27, Linda Sindelar, mackennawarren, MeccaD, Chingea, billy bob joe, loewenheim, jadaelle, janngu651, jaylaowens, Ashley_Garza, aboot4496, Khira, naggii, Aileen_Tran, Jimin, apenr8473, rhill, madd8461, Lily_Hayes, Lya_Carolina, jdjdtx, KylMeaz192, rmurp0900, sarcum728, Carlosss1117, cingvung, JadAla517, Benjamin_Goolsby, Skyler_Riley, Noodles, pdibby, mirledet644, Xx-5P1TF1R3-xX, nsath3050, brunogerussi, Gerda_B., makfos4194, jaume-piera, KCILON, John_Silbernagel, grawat112871273, Sara, AwesomeMode21, giovannigonzalez3273, Stefan_Schweighofer, cody.robinson6, Kw, Mary_Blackard, Rebber97531, a5hm0r, Pat Carothers, mhay1476, kadenclaunch, brox h., giovanabsribeiro, cervu253, mrterry2, kkran4358, Lmcbr9977, MartinVelezPardo, mzdeb, zpxdjft, bramne, gracescorsone, randompersonjci, coryadams117, Neil_Buckley, ALJ Freeman, madiecasten97, sfisher, Troy_Simon, sniel3442, JarSco9732, Moonwaker, Fleur, mnova5686, Isaac_Kleven, Mwmertz, julianagatti, Bonnie_Mohnsen, Madisonreid, MsFidich, Marek, JoyJoymj+zoom, Wfors8718, mieke, opiyo felix lakor, danejawhite, Brycebagley, danielchang3242, GretaB, lprit9442, Rogerelliott50, lweld1635, scalh2319, SoldierPig4, Konstanty_Keda, Robertas_JanuÅ¡eviÄ□ius, EStinnett1, kb, Liudas, FightAlzheimers, moratbuff, Jleung4466, Bliuma, Lilliunivie, Courtney_Coleman, moe113, Katie_Coffey, Dareae aka Dmoney, hmorg0935, shekar150, Ernest, reyesclo, marius69, Sgartewolf, Lazuril, ngask5961, YarnMancer, Allison_O'Reilly, ksmi0434, Irish Washerwoman, silencepain, twintwo62, Ryan_Carney, Triston_Knight`, Reverend_Hongry, boiiiii, gramo4878, John_Durston, kruemel, Kit_Fry, Hamzahk24, Alejandro_Riveros, Jakaria kirkwood, HPGinger, Aishwarya_Kulkarni, vluckila, Mary_Ann2, Abcde, Candice, NancyHAllen, bobbie321, Cindiopia, UnchartedPath, zoli, Domain_Administrator, gbate9824, rued8272, TatiJen, SrCarludio_Kun, Kati, Calico, General, sbrinaton, Chaos2540, Wissensstadt, krara02, WesHargis6896, Astrocitija, NicolÃ²_Pagliani, Meleah Moore, LittleRocker, Rene_Drake, priya_phifer, MommyO, Jessica_Lawes, Tobias, Littlehill, Sgronemeyer, alex_bervoets, gabyv, bah0, ISIAH MCNAIRY, Michael_Wimmer, ALZE, Zachary_Burton, Shiloh123, Ecyrom47, Brigitte_Hutterer, manu_FleischAmFinger, ElzÄ—_RadvilaitÄ—, Xiangru Yuan, gabraylrileysteele, Brian_Edgin, Jesse_Archibeque, ryatru401, Mathew, Samiaint, Biamor388, Monika_MiniotaitÄ—, f_heigl, Brooklynn_Schmidt, W.T., wevrldy, Lan570, AmberSLP, Earthgazer, Ricardo_Garcia, jrodr, colem1224, _Bitmason, Leenix, lindsaywilson, Amy_O'Briant, cassidydavis, Dennis_Ward, Travion Robinson, Zorvyn, ALZE1, chyde5574, keshawnmcgee, Mic117, Mengjie_Zhang, Sky, JohnDavid72, eaw3948, dejourpc-r1, nf, Smegenys, Colleen, cetyre06, Susan_Calkins, Fingula, AdrShad3253, braala112, Chris, maundercoffler, Endurance101, Claire_Wang, Laura_Bradburn, ãƒˆãƒžã‚¹_Tomas, mrsschumacher, Janet Murphy, iron, Vlado, Eelkjef, ahopejones1, Carol_Thomas, rociopuno, Wout_van, Muverethi, Karolis, crimsonanime, LATIYA ROPER, Berutonas, smitht8996, DalTuck729, MeagShow8396, RominaMuÃ±oz, hudabus2, ppalm0752, mommybrain11, Mark_and, MacaMiranda, aeise0441, Sigis, CitSciGuest, rtayl64, Bronx1010, Prince_Lawrence, Samuel_Peters, panchi111, megvos537, Glaciergirl, Jonathan_Brune, Duxan_Arancibia, Otachan, Dr.Mutant, Sharon_Bee, Marjorie_Gibbon, Dtr2Cr31, Federico_Ferro, Celene_M, yyshah102, Sandra, aribell, Trenton_Bryant, superdave50, Evblieve, gey836, Bill-sutherland2027, Danielle_Andresen, jbmwmm, clance, cappawoo, LandonP, ehreece, Autumn_Looney, olivialanier, Mozart1950, emma.rasmussen03, Kenny_Zambrano, dar342, Martincito, FloriG, atren1118, Stoneagemansion, Carsyn9737, kendallnorberg, cmarinaro, gibran.edun, Resalz, mmmmbuti, CitizenScienceProject, Flofis, Rena_Cruz-Horton, KENW, Ryan_Strom, leverimc, Jennica_Bakie, thenickb, Garrettoberg, Patricio_Amenabar, Malik HIll, byazz3188, Lassemiste, Amanda, Doris, seekerdave, jhunt19, GarretBS, Mar'Quan_Lawson, Marili, cawest1994, audreyhk, ariel_lanier, kitkatvw, Support_Team, danieldoerler, abagailcarter, ashlea, snowybeach, Lindsay_Lou, Rafaella_ZÃ¡rate, Kelly_Coleman, avinash_kumar, CeCJaC1778, aicxoxo, Shandon, badeyes, Anastasia_Kokori, pb, Nozomi, Michelle_Neil, Danuta_Ciesielski, stuartes, opit, zgoet2214, rawlsy25, Amy_Edwards, xzhang6, AllSci85, Susanna, OIS_SHS, deon reid, Derrico_Carter, sophielehner, gameon_user, JakobFSNF, pnau, icook1093, dmcca3433, greenjeans,

Joseph_Taylor, Malu1525, evanfunky, John_Pearson, Green_Furry, Jayden_Eastman, J_Reulen, olga_garcia, Melmcd, joshsf, Kasia, Catchthatstall, Roger Rasmussen, voltmtndew, JMH7167, AZAmes, theyseemestallin, Oskari_LeppÃ¤aho, isabella, carolmswiz, orla, beckseb, Catalina_301, elizabeth.lopezperez, darksagan, Viktor, rming, Jacque.blundell, harmonious, SarahWest_SEI, Marax_Custos, kbears13, Kmccary, MikeC, Jens_SchrÃ¶der, JiDaYe, acpratas, noelstoj, adavi5295, KELLY CORRIGAN, srosi1949, lburg0544, Michael_Huang, wideawake42, spamt, johnsampson, Boomer, valenciasegura, Lyseblake, Julian, mroberts, David_Green, RamMarte, Alzres, Kimberly_Hoke, nyasiahawkins, GameOn4, Tatty21, Lvl1savage_Boi, Joyce Digges, ellbarn3736, BaileyLyman, Aaryn2018, Aljereau_Marten, TripleGold, InventorOfStuff, Thomas_O'Reilly, Anneeb, tomh, David_Colborne, Chuckayl, annalisecrockwell, yvonnr, Ameal, rmart2146, seanflynn1985, Antonio, jaylen Greer, joko, mohamadreza1999, csp847, Disha, WendyD, criscapiner, AntoniaC, Lukas_Saduika, Chase_Hosbach, LoniCronin, Jbarn0008, Tomislav_Kos, MSAR08, jkakodkar, stewbomb, Isaac_Faller, Mary_Jo, Gwen, Joann, John_Vinarcsik2, jamesdeanjr, Rudolf_S., Auvrelle, Changchiayu, hillrip1, TheGymnast2001, kln71200, gjcbrain, chartreise, Diego_Silva, Justasnor, Deligreu, Gea, MomsBuddy, wendywang, asatterlee, Ksalerno, Catherine_Tyre, Linda_Fordyce, j_Elizarraras, JFulks, introvert_dave, saina_zarrabi, mirgisnat, umut, VArrington, Tinina, Dustin_Bolduan, Krpritchard, VirginiaNichols, HaileyJo, menantol, Faith_Newton, Jessie_Oliver, luizgualmeida, Maxi, jvernam, Shimon, Kpruitt, RITANSHA_PRASAD, SaraK, Ecro, Karolina_SavickytÄ—, rhiannadecleene, Livius, FahKai, egash1355, sgollapudi, rbeus0334, ddavid, Yorkie, gwoods, Joelle, Mary Gregory, Bukhover, Lisette, Karen, rahmeh2008, carlos_villalobos, Star_Warr1or, Catherine Scorcia, ybhatt, George_Halkias, JuanLuna, MarÃ¬a Isabel DiÃ©guez Morales, David_E., Hayden_Carter, inuss7151, Karenbaker, ashleythompson, ironmanstinks, ptower327, Aftermath, Amanda_Boling, Jmm119, Jideobi ujnnwa, Brian_Loughead, mmukon, cman, LyndieLu, josephcantwell, Kestrelqueen23, Hunter_Phillips, Valentina_Abarca, josiahowen, J_Crisp, Christen Sgroi, Glitter, Julieisprobablyalreadytaken, larry_trefry, Ljkinzer, Debgies, jon.k, Linkess, Devon_Gere, Marian, dkamp, crystalrussell, efrankus, rumptidumpti345, Benjamin_Garrett, Crench, Natalie_Zaba, Cindy_Carrillo, Semperfi0341, Miranda_Jones, Bethdt, OwenPriest, Shyannah_Frenzel, Ignas_KanÄys, pel, Gavin_Clarke, jpbennett, Rigo, Ian_Amaya, Hermione Granger, Max_Yam, howardhanry, mguertin1, SandyBowers, Michael_Sutton, Gabs, Katherine_Linn, lboth0364, Gwen_Jones, Margklein, nafdarb, fred.slota, Joel_Robertson, gearhead230, Jaelynd_Wilkins, Dualyn_Hill, Robin_Bennett, PhoenixFoo, rtshi0713, kp544, SherriMiller, aappling, Zach_Marcovitch, aross1, Scott_Shaffer, Joannie_Aiello, Dave_Desrosiers, Carolin_Briganser, Ranipani, rwads1397, roy, Dmrs5828, Lidia_Garcia-Campmany, tyleegunnell, hcall, Janell_Webb, crobinsoxnd, BillMitchell, Virginia, Nicole, colincarstairs, Simeon_Langer, Highperion, Anusha_Swamy, Rachel_Stevenson, debbly, sombi2, Melyna_Lemon, Grzybmm, Victoria_Medina, Carly, Moaub, m.rugile, JameirConley, leahwarren, Kayaman, Christian_Schlager, Kkamerin_Fair, happycolourguy, Mike_Heron, tjohn5348, spetersen, joplatt, ebixl0342, findacure, Aprez, Mindaugas_Kasiulis, seanmcdowell, Abel_Pina, Augustinas_Giniotis, wvh, appe001, sashaheywood1, NicoleB, ebates, Etecon13, Christine_Rybar, Katie_Thekleeninglady, vcoope, ianmac312, zmallon21, davebelden, cbemis, rbagge, love4all, dtcstall2017, Noah_Waesche, Janyawoodley, Giedrius_MeÅ¡konis, Gameon3n, jaya, Kayla_Goodman, thefivepoints, Andrew_Cassady, conjep, hasmodius, mckennaray, Messeguer, Mike_Scafidi, rkaisler, BabsUNIVIE, Larissa_Ann, tnorw6683, Nada_Ata, joeijoeI, lou, Zack_Korman, Rdanie02, EthanSanders, brooklynanderson, Stan, kpolczak, cockeyl, mphermosilla, Rewindbkind, keona mcgee, Gerimantas_Ribinskas, Matas_Lecter, SteF, Airina_SalytÄ—, YiOu, Jennifer, hk, jpell64, Dukiemac, Dj, Keiko_Nakahodo, bettyboo, CaptainSofa, Ryanaberle2002, Bojan, Barkley, CPwdA, svar, MarieT, tlewis, ESPoppelaars, DAV John, Sherry_Keener, Bexu55, kkmdj, Sznncarr, klaw1034, ckibi2006, nishant_tripathi, TERRY, AdaZach, Desertdew, screamingredrabbit, Rio.reeves, aikawahigashi, jacob.andrew999, ZGWZYER, Isaiah_Fulton, missmomo, kaylawigley, Karin Raab, Jiannan_Che, 300rmt, merlin5353, John Erb, Robert Larimer,

BlackDoily, stoensmann, kenna03, OwenMRobb, petersonjp, Conner_Gifford, jodysworld, Alexus_Chua, ccody, Steve_Smith, Yoni, Brock_Bindrup, DiegoPelaez, Sheryl, Zackery_Parker, Charles_Motley, Camshell, Anne_Bottcher, Merida_Aynat, Gilberto_Alas, Philipp_Rauscher, Sheryl Teti, neuroflo, J._B., la_jovita, eusgfo, Vladislav_Belozerov, alex686828, julsbur1, aphasia00, Bekissel, shulark, ValleGirl, Geobellward, Andibelle, Lsto, nathanialdcook, Haliewood, COOLBRISAZ, Tbone, eye, Alice, DaisyTET, Isabella_Ford, mkdouglas52, Susana_Artigas, Jeanne_Bedrosian, bradkuhn, INDIA MOORE, mr72122, Madison_Larson, g0147410, Matas_MatijoÅ¡aitis, Dorothy, Andrewmeyers95, george, CDAlaniz95, Lydia, Jasmine_Loo, Dhirendra, RichBroth, missyegm, p_guerrero, XtraDad, elchenoweth, JazzyJuarez, aliabushanab, Brenner-AdamsR, DeirdreMurphy, Victor_Linares, pwann3490, Raven_Sansoucie, courtneycandc, gingerella, AT, miiichiii, SierraMikeZulu, Weaver214, kepps0796, Harry Potter, juozasmilius, Stingaj123, Chukwudubem_Nzewi, Cydni, areev9982, å¸Œç¾…, BreezyDionysus, colefri63, bio1010, nhoaldridge, Matt_Yang, nwalat, andy-p, SBrough, wwilliams, cjsmom, araytiay, Natasha_Papke, ZachKwedor, Nlgeorge73, IFarrell, chuckdotson, Alexis29, Robin, Sandra Marroquin, Kristin, Erik Lehmann, J.J., Pakyuen_Lam, Nicola_Butterworth, Tyler_Garza, Tom_Pui, jjjjj, tesselarson, ehamelin, Vicki, Chandelhilarydanae, Fahm1, lindamack, Richie_Peters, Miller142623, emirose333, Martin_Zikmund, Vicky, Jsacchetti92, eghalpine, Bonniesfcc, NettieStevens44, ahoero, Deivydas_Bakanauskas, ng, Anitha_Manohar, MHenriksen, claudine.trottman, Nonsochivalrous, Barba, _Martin, magalinskies, slnu, SonWukong3005, rturmel, Tim_Dolan, Physdude, JimmyDabomb, AjKhandaker, Jaimie_Warren, Aaron_Lewis, ShadyKay, javl, Pigloo, Rachel_W, MarkJohnson, mrn2017help, Marcivi, notasissy88, Hanako, Ines, autumnsmith, Malanahhasan, amandaburks, alexklar, Alstall, kale, xtayl4856, Justin_Wang, 14-0127, Gillyboy31, Aurora, alinaja, Nickb728, marygemba, ReemGhabayen, Yusen, smile9966, Sir, Dayman, sammybrink13, AndrewSlater, jausmh, Michelle_Wall, NurkRodz, Rodrigo_Bernardi, voltairine, wmchef1988, Holly_Deal, Michael_Knoll, Kateewall, k.conover, Allison_Semaht, JBMuller, braedobudge, Asome7_5527, RuthB, Wilfredo, Brandon_Hornsby, finape, AdrianaP, Mlwfee, kirschenjojo, Daniel_Carranza, TxNx, Kevin_Chen, sastorung, lam, kelseymarsh, eforcke, Flantier, aclab, kriff, Lindsey, nnicol, Barbaraknecht, pamplemousse14, Kara_Maynard, DeAngela, theeverettclinic, Tosha_Windsor, Mihaela_Rosu, jansrosin, Mark_Julie, Judyjohanson, Bryton_Harlan, AnnetteNabirye, Basilooton, Barbs217, gertswerks, aaronbyum, reiterhelm, mammaroni, mat_e, pershler, Sophia_Melendez, Chelsea_Ridenour, Marji_Buyea, bethanie, chorvath2000, Carly_Pherigo, hipple17, Danny_Wei, Porkytail, sydcrook, loopology, Jimmy_Du, Tami, Jennifer Samsal, mmaccara, helloubunnylovers, tfree5593, Palmer_12, Kadeo123, sariecatherine, asa, Cwmensforth, Daniel_Burke, Wilecat, va22046, snhogan, matthews33, Timothy_Morris1, azpeg85, Vilnius_Ramanauskas, Ian_Baxter, anakin0305, lf375, noah schmidt, M, Emma_Cronin, Lucas_Linn, Amanda_Weiss, Bolarinwa, fuerholz, Tamara, eggling, Madeline_Harnstrom, Jennifer_Adams, nfors0627, alaylayTharp, Jadyn006, oliviajames16, Kelly_Biddle, msloa5321, olliewolly, lispleasant, marilynbetanzospetersen, Hamgirl18, Michael3456, Eric_Knapp, kpiippo, Arlene_Peniuk, larsson.emma97, SNLVRS2, Grovemarg, dlteare, monroviabiotechday, SharonLSinatra, Jim.lawson, rdiroll, Threemurrays, blewturtle, Sri, tja, 500Z, anns21, Gwhdah, Moonshine35, Turing, daniellepike22, Lynnette_Wissink, Sarah_Rae, jitssssss, jameswinder, Lins, Brittman, Tiffanylj5, derrickjones, Claudio_Ehr, Dasha_Svistunova, Ty_Lawrence, Jayden_Youngblood, Lance_Strahan, mazoola, Hayray13, Alexander_Jackson, Melissa.J.D., Candle, Mariakuda, Morey_Hallett, Mozfest2, Mic_Lin, Chi_Chen, carlyng, æ¶‚ç‘‹éºŸ, Nadia, caitlynhowe, bfarrell18, Robtheblob63, AnnaNoelStorr, zfors0489, heller, jamgearhart, Ninchisho, ilaz0598, teejohns5, Bensprouse95, Chris_Dolce, hazletta, Tulle, TinyT, Ioana_Cristescu, gardengirl, brooks.kelsey14, chanbiedermann, kristinhodge, bflint43, MDay32, abbahartle, Carolina_Cabello, Becky_Camenzuli, Jacque_Harris, Tyson10130, jbdpgyt, LM, fabiobruno, djtulop, cornflower, sztak, Vaida_LajauskaitÄ—, Grandma_T, Amy, Bob_Johnson, mattom75, kitzmiller, Alzheimer Filial San Miguel, Gamberb, LBG1, suryakarthikeyan95, GameOn2, Dizzmas, RimantÄ—

_ÄŒirvinskaitÄ—, Nojus_ArbaÄ□iauskas, girdyatsea, learehman, juliag, TheTechnic_Lord, Sschot, DataDan7, Artywarty, Lina_JakubkaitÄ—, zwelsh94, morgun, Mangam, Emilis_KiÅ¡kis, Michael_Ohl, Hwb4146, daniel871026, kaguya, fucitaszole, Joanna_Pang, Dartha, RobertG, Leila Aryan, AlexandraMca, LauraDN, iamemalym, devynhebert, Cindy_Larkins, pumpkinbumpkin, CalamityJanet, jnine, Andrew_McConnell, scowsh, CWinder18, Jenae_Howard, KotaAzul, Lprongs, kingman, teebud098, Dave The Drummer, oateasse, Gage_Thompson, Seven, Gonzalo1984, Emilie TESTAN, gsamd, LyraStar, JMorgan919, Radvilas_Bendorys, Ryan_Akerley, jasminejames, Noe_Mijango, GameOn1n, GameOn3, Am42, ejm_alzfighter, Sydney_Langer, Bryan_Miner, colbnels2023, anaskye, Douglas_Cardin, Marius_Paleckas, zfoxt9255, Kasie_Keene, Kes123148, Damica_Clerveau, Brandi_Elizabeth, 1989akame, Max, ken2.0, Mumijump, Rebecca_Chittenden, Kimberly, ereed, Don Lindsay, Alessia_Viano, jgarc0196, Danyan, GreenSnakeLady, jwestwood6, Manjusha, janicelee, Akairborne, JKBROPHY, Cshimeal, é™³æ€¡é›¯, cuterocketeer, hunterl, speakeasyangie, lizbrisc, NicolePellegrino, Sydney_Jones, Lizardesque, help1234, Wardenweeden, justeengonzales, MylesS, mwithers, caseyrfriedman, xhojyrj, NateIsBait, gboisvert, Emilee, Trump, Jayden_Smith, Derek_Parker, Bongos, JESkaley, Meggie_Tseng, antonio_stall, butterflyZ, maysynharris, nicole8228, Brollypower, GameOn666, CK, mrondina, jscantu, Destiny_Denning, Sarah_Dearman, James_Taylor, Tyler_Noall, Leyka, sharliit, vmile5525, Spooky.Yanna, Mozfest1, lauraobrien200, littleme, Michelle jou, Wenchi, NickCicero, Ctornquist, jadenn, AJIsLit, michaelli, B0621146, kftina8, jaredrmiles, laurenthom25, Southampton, STEMlover27, mrf, Peter Scanlon, SnapDragon, MissKelly, David_Johnson, Jake1028, jms2313, Prateek, Claudia_Covi, RohitSatyam, Sharkman, kellyerin, wjtn, Heatherlindam, Alison_Lee, Christine_Wang, Suefitzpat, Stewbie, taylorm9414, AshGBell, daisynavidson, Archer_Northrope, BethP, aanderton, CassieWilker, s9357, coralclark2001, Samantha_Aiken, kassandralcole, QRXTS, MN2011107694, Suzannej30, MalÃ¨ke, elBirdo, PetersVolunteer, Wdrollette, jklackl, lgreen527, lwbarcelo, contrapost, OrionBell, Marija_KurklietytÄ—, Lisa_Lechleitner, pascorandy, Liz_Evertsz, Chris_Sturgess, Lori_Atkin, rbdanley, Jurgita_AliÅ¡auskaitÄ—, Da_Sisnie, DiamondDog42, Jlyell, Jeff_E, adriana_delgadillo, ahunt2083, naominumoji, awagn0860, Gary_Low, joey, Wang, æž—æ”¿è«º, Suga, M.Zare, fedebarru, emba999, cathleenvoelkel, davidfigurasf, 118736c, Boja_StrazdienÄ—, Ieva_KvintaitÄ—, Ryan_Dickey, AndreyMpr, Phallquist2, cCHELL, roland, Zamojuni, mfrei0631, laterleigh, pzaret, sbtanner, Maquel_Hoover, Grant_Smith, Nol4nJ, Davis_Madsen, Maryclick, Spongbob, kjeldmooi, Omar_Farah, Molly_Brown, Angelad, InHonorOfMary, Roberto_Mascia, Mbs, LilliLover, BarbaraZ, Suzan.seifert, Lost, Frankie, 'Rik_Smoody, Tabootatt2, Chuck5354, Sacha_Nichol, Chung_Tsz, Natalie_stgermain, Melanie_Batarseh, Anja, r.b.taylor, McKenna_Wiltbank, Elaine, Dallon_Schofield, tschingtschang, Tim_Reichert, Twinkie_Chan, IvdM, Revvia, Fabian_Platz, EZE, Wyatt, Malorie_Kimball, worleyr, amisus, Germ fighter, Maudie_Bales, gcall9421, Elijah_Wilson, Frumence_Kariuki, Luci cury, AmberRose, Sarah_Chapman, Gaetan, Zariajackson, chadsitysmith, Jayce_Jones, grue, aukseka, Silvia_Arevalo, Dore, Ema, Aracely_Navarro, Charios, Ingrida Leščauskienė, James Wilkinson, Darian_Kearns, Bobafotz, rcacciato, Christina_Cross, Sebastian_Recce, Elaine_Halley, David_Rowe, Sciencehelper, eschn7969, DaAmo, dani, tport0439, Dodo_Ke, Barry, lucas861223, JIa_Hao, RA, Engineereric, 29347322, B0544147, awood, tylerhatch2, Brionne_Vinson, Windig, dilek, DBGladden, Alexander_DÃ¶ring, bentleeh, Dejahlm, Robin_McKenzie, AnimePersonRylee, Griffin, Charlotte_Levy, Laci_Waters, Mary_Williams, Pyro761, bunniesareamazing, Robbie, ctidwell, camie_rae, Phoenix8899, Lauryn_Eppich, stephaniecarpenterr, Ashaw, RiLoosli, sammigangloff, ktabbert19, Alexa_Johnson, veterinarian, cherylann83074, Ed_Jaworski, S.AKASH, Michael_Shoop, Juan_Luna, Msumuh, scorpioneye, rgbrown, Michael_Strong, Jennifer_Montigny, pattybale, Miranda_Knox, kevinv312, Lindsey_Jacobson, Kathleenpgarcia, lsdfree321, cunnashlea, maldavis, cewever12, zachsurdell, jalon, megan.wilson, lhouskeeper, Patgirl, slagter, ryanmlock, Selgthered, Melgomez8, Sarah, samantha.taggart, jlwalker, Tfulls4, Catch22, Streamckinlay, London_Crandall, murdock59, Jack125Clark, Sharon, Monique, knolan19, Sqbleau, carmen, Simei, Cpa, Alissa_Thompson, adraper,

nana corrigan, Dinka_Lac, foralz, Andres, kaylavasquez, SehajChand, tradynrandolph, jeremiahmartin, jadenbuford, tajunajohnson, Carla_Amaya, mvolmar18, Martynas_Alekna, Tygeris, Neoliya, Kristof_Vogt, Pegff, aditi_B, Sebastian_Melendez, Jim_Manley, yangscleavage, DavidHIL, socalboy, GEM, jacobb, cookm987, Ieva265, Mario, HeatherB, Karen_Dugas, ccari1572, Mac, HaruAndAki, Jordan_Delisle, Liu_Pei-Chi, aarquie, Poassac_Hsu, jaasstin, Jay53, Andrew_LI, Manos_Kelaiditis, A11red, kbolton, Celina_Sta, Derek_LeCheminant, JacobH99, jakebhunt, rbraman, Ocelotarian, Jimmy_A., erik roge, Xzander_Taylor, jmart9088, Ziggy, overthere2, jaycie_roberts, jacecox, Rebeccaturner654, Ohemaa, nikki98, Gdaddy, Derek_Richins, bowers_m, cole.utefan, Wyatt_Norton, ezak, Stevee, kylemcd12, Monika_Koblak, Baller94, Piper249456, Janice_Blum, Mr., Lydia_J., jip111, Lornaw, marygreggbyrne, JWAshbaugh, Carly_A, Beverly_Wiegand, Nurmit, actiasluna, astan3936, Gina_Lynn, C40142, Harleyhoney4u, dhammvt, azurefire44, Andrea_L, Haritina, Heather_Burkitt, Raoul_Rosenberg, Pam_Sprute, Sheba1, Ann, solsultan, Amanda_Fraley, Sydkid915, ghilbrae, Sahil, natalie72302, Benmorris32, dstevens21, Abby_Kilpack, BalakayB, nathan.swainston, alisonnntanner, Joey_Menichini, stroudies, Anastacia_Isamar, luckett.kim, kc5nvj, naveh, dmarks, veganmua, AAAAndrew10, tsjens, Victoria.Saunders, Savannah_Argyle, Meggane_J, BuckyBarnes, Averyfd3, Goldfish306, Chey08, kiyanoosh, Jesse_Chafer, Denis_Robichaud, tarasank, vmuse12, wizzwizz4, SUKRIT, Jacqueline R Aviss, Parker, kvorther, Vicente_Breton, Yerko AndrÃ©s, soununo, Kittydoc924, Cmykitt, Hyunjin_Song, Abby_Justiniano, Marko_MÃ¤gi, Drbrian, Linda Rinehart, 19manigbehb, laurenwigley, Jonas_Motiejauskas, Gary_Tam, Gediminas_Braskus, Danny_Stenavich, Kaitlin_Welsh, Garry Anderson, Cierra_8, Tyra_Weiss, Navano, Elizabeth_Graziose, Pdole, DangerMassimo, HelpOthers, SparkleHeathen, Elysse_Ciccone, rudal, Vytautas_Liu, Quinnc1948, Taball123, loysjohnson, nat4hagen, Giedrius_MaÄ□Ä—nas, Danila_Maltsev, Garrett, rumph6350, Jokut10, carmjon1, æž—å®¸å®‡_109ç´š, hanyun_liu, Stephen_Kerns, Meow, lukepierce222, cafemon, AKaranges, Jacob_Siu, hannah_kay36, igonz8206, a97410985, JamesB0623045, william31525, CharHart, kassikunz, Claush, Maria_DiDesiderio, Amy Ward, JordySpethers, Natalie_Putzel, Connor, loganj, drewm, LittleFoot, Marin Miller, Michele_Massironi, JulianLang, Bonnie_McClellan, Danny_Richard, ladykatykat, Jesse_Ballenger, gsmithtx, Ttbrow2, _liv_lee_, smcfarlane2006, Deanna_Dickmeyer, dwiberg, si09, Duaa, Jamieroger, jessikajenson7, hillmsara, ascot9364, chriscalla, amblake, Susan_Brandon, Jackson_Turner, blakenelsonjordan, Nancy_Ruiz, brittadams, aubrie.saetrum, kylerhack, Adam, Kyle_Morris, StallcatcherUSU, TheRainMan, Obeebe, sarahnewby14, Benmiller3, Preston_Hall, Laura_R, Markypoo1325, Allie_Sharrai, kafann23, abagelly, jaxomrosenlund, Kayla_Ruud, Alyssa–Redford, Kameron_Laird, kellen.gillins, JasmineTurner99, haheather, Lauren_Beckstead, lesliegm16, Kade_Jorgensen, janine_hauser, scout, Rian_Dowd, MutatedOrange, Portgas, rbeachem, mbritsch, chamm, camzand, egrowney, scoc7007, Liverpool88, Settlechaos, jone7916, Jg, CRahm, Kayakkid, Lee_Hartsoe, gpelle4, friedii_haube, nvannelson, Jared, BC, hsung, alaia.sb123, Katherine817, t.deese, cdutson, Akramova, Ashleigh_Bope, wollito, Jenna_McMahon, kbradle6, Tylerlloyd30, skas121, Avery, merabmcfarland, 12snow16, samwright, Catalina_Harrison, kermin, Emily_Gordon, Ijustwannahelp, jdavis41, randerson, AlaunaR, voigtlo, EmilyRuss, Blueplates, cduff, jimaker, Pia_Z, Mrs., 1starfish, Carlo_Toniatti, MsSalli, Mairi_Breslin, elleb123, Moxie, skittledog, fjvarro, Sethanon, Sofia_Villafana, masonjb, X3IV, justinalder10, Preston_Barlow, Awesome, Shane_Stroder, mattwoody, garrenburgess, Landon_Rasmussen, Vincent_Olsen, Hillhall25, ArFo, Prairiegal, samiel2012, kbruffett, Madisen, LeeAnn_Crittenden, coloradojed, Gabrielle_Hebert, Mom, Colby_Pride, Carl Landers, Kysa_shay, dragonturtle, mrippen, tcroc0240, Timothy_Kulp, Tyler_Cartright, Cassierobinson1, Shooter35, IvÃ¡n Konar, Rosara, Drew_Ferrari, eileenslauenwhite, Tom_Tyler, maggieauger, Martha_George, dolina, marcuslawson, 18nevaehag, 21eliyasb, Tanja B, keshawnmo, urtetamu, Siuyee, Christoph_Eberl, Stefan_HÃ¶ller, Zibi, Bef, Julija_Swan, Michael_J, Tkleggate, Linda_Fuoco, Bryan_Hamel, Gavin_Harmon, Lbartosh, Cafe74, Cindy_Leung, Tulikaj, ryguy94, bionicrack, thomahawk50, marina1127, Validator, KadenC, keelyrae, bayh, Existone17, jadki0414, acook0987, Sarah_Zuhlke, Yvette_Zhu, idowu_vincent, Dream5089, Michelle Huang,

stev2125, Duncan, kasmir, Joyce_Yuan, erik1994nl, pip69, amart5414, aloha, yohane, Rong, B0629014, Corey_Neskey, liv.llama007, sandybrown, tayap77, Navar22, Rvanderwerf, birdmarlee, taytaypippip03, Zallen72, Angie_Kays, Julesv3, allyvalencia, murrayp, KeilsNicholas, ikerhea9, chealseymarie, aotearoa, Shannon Heintz, Rainer_Holzapfel, sking, kcsj7816, Catharine, Geruta, Isegrim, Glenn_Parker, bsh4000, uellis, Shreyas_Chawathey, omni2005, Emmalee_Clark, marci1181, JBaxter, wethada, JSoup, Cali_Hollie, ekkonightshade, jwood25, Dallin, Tawni, KateKid, EHeaton, Odang21, tannerlmonson, mercedesmickelsen, landiony, dainevans, lizzyconto, Jake_Hammond, mistys16, Peter_Call, Madison_Hawkins, karristru, baileygirl3, alexisbri, yomotwin, jonbritt182, Helyosprime, loulou99, spraktes, jocelynhartley8, alyscoffield, macmulthegr8, Abby_Harris, campeck, super3508, ehatch, musictherapymanda, Nadia_Nasah, somethingorother1234, mr.awesome, Potatoeaim69, Ching, vmanjunath, Trash, supadhyay, Vanessa_Ross, YourDad, lilithslays, Ammon_Dillabough, Climberbri, scprobst01, tjbauserman, caitg789, Cloe_Carlson, damienne, TdArtenay, Texnia, analeagardner, VermontMade, CtheVet, Ariburnham, Shonigman, Eyenwang, Moyer1948, Bernhard_Gerl, Anilecia, Jamie_Williams, Elinva, Spardova, forge, mettmann, Evilpegasus, bvasquez, chickenbug, ccarroll3205, Bobdaloia, walkingdrunk, alindseydecker, Steven_Voyles, Kridder08, Frank65, Ljheckler, Pallie13, amf31892, ashton03, jauz, pittypat, Will_Roberson, lythgoe27, Coobear71, HaleyFishy, Stephanie, jpatterson, jjjphd, tolivi6, kwikitti, Mark_van, doggoclan, jhormd, rashlyn98, mtaggart, Ranjit_Ramachandran, Scott_Murphy-Neilson, Aaron_Johnson, Lars, adrihuyghe, owenlinares, Jeffrey_Deal, oindrila25, kommandant2
